# Yielding Elastic Tethers Stabilize Robust Cell Adhesion

**DOI:** 10.1371/journal.pcbi.1003971

**Published:** 2014-12-04

**Authors:** Matt J. Whitfield, Jonathon P. Luo, Wendy E. Thomas

**Affiliations:** Department of Bioengineering, University of Washington, Seattle, Washington, United States of America; Johns Hopkins UNiversity, United States of America

## Abstract

Many bacteria and eukaryotic cells express adhesive proteins at the end of tethers that elongate reversibly at constant or near constant force, which we refer to as yielding elasticity. Here we address the function of yielding elastic adhesive tethers with *Escherichia coli* bacteria as a model for cell adhesion, using a combination of experiments and simulations. The adhesive bond kinetics and tether elasticity was modeled in the simulations with realistic biophysical models that were fit to new and previously published single molecule force spectroscopy data. The simulations were validated by comparison to experiments measuring the adhesive behavior of *E. coli* in flowing fluid. Analysis of the simulations demonstrated that yielding elasticity is required for the bacteria to remain bound in high and variable flow conditions, because it allows the force to be distributed evenly between multiple bonds. In contrast, strain-hardening and linear elastic tethers concentrate force on the most vulnerable bonds, which leads to failure of the entire adhesive contact. Load distribution is especially important to noncovalent receptor-ligand bonds, because they become exponentially shorter lived at higher force above a critical force, even if they form catch bonds. The advantage of yielding is likely to extend to any blood cells or pathogens adhering in flow, or to any situation where bonds are stretched unequally due to surface roughness, unequal native bond lengths, or conditions that act to unzip the bonds.

## Author Summary

Cells adhere to surfaces and each other in the presence of forces that would easily overpower the individual noncovalent receptor-ligand bonds that mediate this adhesion, raising the question as to how these bonds cooperate to withstand such high forces. Here we show that cooperation and robust adhesion depends on the elastic properties of the bonds. A type of nonlinear elasticity referred to as elastic yielding ensures that the total force is distributed equally across the individual bonds regardless of geometry. In contrast, with linear or strain-hardening elasticity, the bonds that are stretched the most are exposed to higher forces, which cause them to fail sequentially. This work explains why elastic yielding is found in structurally and evolutionarily diverse adhesive complexes.

## Introduction

Bacteria and Eukaryotic cells must resist mechanical forces when they bind to their surroundings. For example, bacteria and blood cells adhere to other cells or tissues in the presence of fluid flow that applies a drag force on the cell, while many other cells apply force to each other or to solid surfaces via cytoskeletal contraction. These mechanical forces affect the lifetime of the individual noncovalent receptor-ligand bonds that mediate cell adhesion. Some receptors form slip bonds, which are shorter-lived with applied force. However, it is now understood that many adhesive receptors form catch bonds, which are longer-lived at higher force [1], [2], [3], [4], [5]. Still others form ideal bonds, which have a constant lifetime over a range of force [5]. However, all bonds transition to slip bonds above a critical force, which is generally much less than the total force involved in cell adhesion. Thus, strong and stable cell adhesion requires clusters with multiple receptor-ligand bonds. This raises the question of whether cells have evolved mechanisms of stabilizing bond clusters.

**Figure 1:**
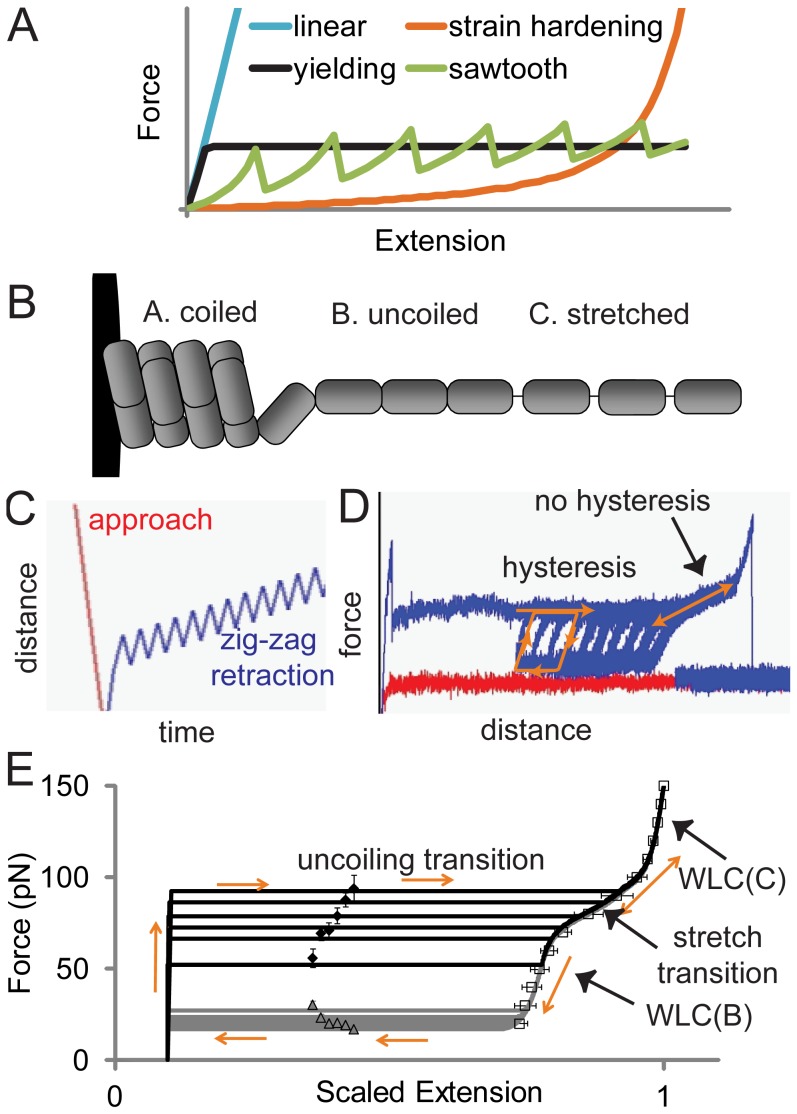
Elastic yielding of fimbriae. A) Types of tether polymer elasticity. B) Three states of the type 1 fimbrial shaft included in the model. C) Movement of AFM cantilevers in experiments to characterize fimbrial dynamic elastic properties. D) Behavior of fimbriae in AFM experiments. E) Comparison of fimbrial mechanics model for extension (black lines) and retraction (gray lines) to experimental data for extension (diamonds) and retraction (triangles) forces and for the shape of the post-plateau curve (squares), for a range of extension and retraction speeds. Error bars on symbols represent standard error of the mean of at least 8 measurements.

Multivalent receptor-ligand adhesion is affected not just by the properties of the receptors, but by how they are incorporated into a cluster or cell [6]. For example, a receptor-coated and a ligand-coated surface can be easily separated by peeling forces, which stretch bonds to unequal lengths, but resist much higher forces if all bonds are stretched to the same length by shearing between two parallel surfaces [7], or if multiple bonds are stretched in parallel [8]. However, many surfaces are rough or curved, or tethers have unequal equilibrium lengths, so that bond strains are unequal regardless of force direction. When bond strains are unequal, the elastic properties of the tethers anchoring each receptor or ligand to the cell or surface affect how force is distributed among bonds. For example, longer tethers increase the rupture force of the clusters [9].In most studies of clusters of bonds, it is assumed that tethers are either stretched equally [7], [10], or are Hookean springs [11], [12], for which force increases linearly with extension (Fig. 1A).

However, many biological tethers anchoring adhesive molecules exhibit nonlinear elasticity. While entropic polymers and tissues often exhibit strain-hardening elasticity (Fig. 1A), many bond tethers exhibit yielding elasticity, where the force plateaus at a critical force, allowing long extensions at a constant force (Fig. 1A). Yielding elasticity is observed for biological macromolecules and organelles as structurally and evolutionarily divergent as membrane microvilli [13], [14], [15], [16], alpha helical proteins [17], or quaternary helices in bacterial fimbriae [18], [19], [20], [21], [22]. Moreover, the ‘saw tooth’ pattern (Fig. 1A) caused by the sequential unfolding of multiple globular domains in proteins like fibronectin [23] approaches yielding elasticity when pulled more slowly. This raises the question as to why yielding elasticity is so common in cell adhesion.

*Escherichia coli* bacteria with type 1 fimbriae provide an ideal model system for studying the role of nonlinear elasticity in cell adhesion because the adhesive structures are well characterized. Type 1 fimbriae exhibit nonlinear elastic extension due to uncoiling of a quaternary helix of a linear polymer that consists of hundreds to thousands of subunits [19], [24], [25]. Each type 1 fimbriae has a single FimH adhesin at the tip [26], [27], that form bonds with well-characterized properties [2], [28]. Depending on the FimH sequence, FimH can form either catch bonds that require force to be activated or strong slip bonds that do not require any activation [2]. Type 1 fimbriae thus provide and ideal system for understanding the role of tether elasticity in dynamic cell adhesion. This would require methods to probe the forces on single fimbriae during dynamic cell adhesion, and to change fimbrial elastic properties. Fluorescent methods don’t provide high simultaneous temporal and spatial resolution, in spite of recent advancements in fluorescent force sensors [29] and single molecule fluorescence [30], while other methods of measuring tether forces [31] disrupt adhesion. We also lack methods of genetically or chemically altering fimbriae to dramatically change elastic properties. Fortunately, computational simulations [28], can be used to probe bond forces and control elastic properties. Adhesive dynamics simulations were applied to *E. coli* binding via type 1 fimbriae, but yielding elasticity was not incorporated because simulated forces were too low to uncoil the fimbriae at the flow conditions studied [28]. On the other hand, computational models of uncoiling fimbriae have been fit to data [19], [24], [35], [36], and used to predict functional advantages [19], [25], [37], [38], [39], [40], but have never been incorporated into experimentally validated models of whole cell adhesion, so the importance of fimbrial yielding to cell adhesion remains unclear.

Here we use type 1 fimbrial *E. coli* adhesion as a model system to investigate the role of yielding elasticity in biological adhesion. We develop a complete model for fimbrial coiling and uncoiling in dynamic conditions by fitting a biophysical model to new elongation and contraction data obtained from Atomic Force Microscope (AFM) experiments. We introduce this model into a previously validated adhesive dynamics model for bacterial adhesion without fitting any additional parameters. We validate the complete model with new experimental data on bacterial adhesion; both model and experiments showed that bacteria crept forward but did not detach with large increases in shear stress. We showed that robust adhesion at high flow requires yielding elasticity since it could not be reproduced when other elastic tether properties were used in the simulations. We analyzed the underlying mechanisms to determine that yielding elasticity allows a nearly perfect distribution of load between bonds that were stretched to varying lengths. Finally, we predicted based on the simulations that bacteria binding via catch-bonds can withstand low flow only if exposed previously to sufficiently high flow to induce elastic yielding, which we validated experimentally. These observations demonstrate that yielding elasticity is critical for robust cell adhesion in dynamic conditions via noncovalent bonds.

## Results

### Yielding elastic fimbriae model fits single fimbriae stretching experiments

In order to characterize the elastic behavior of fimbriae in experiments, we stretched and relaxed single fimbriae in a back-and-forth manner with an AFM (Fig. 1C). During initial extension, the force ramped up rapidly, then plateaued suddenly (Fig. 1D), showing the instantaneous switch from linear to yielding elasticity that has been observed previously for many fimbriae and pili [19], [24], [35], [36]. During the back-and-forth movement, the fimbriae demonstrated hysteresis since the force cycled between a higher force during extension and a lower force during retraction (Fig. 1D). The force levels during extension and retraction were calculated from pulls on several fimbriae at several velocities, (diamonds and triangles, Fig. 1E) and this was used to fit the parameters that determine the transition between the uncoiled and coiled states (*x_AB_*, *x_BA_, k* ^0^ *_AB_*, *k* ^0^ *_BA_*).

After a long extension, the hysteresis ended, so the extension and retraction phases converged into a single S-shaped force-extension curve that ended at about 150 pN with detachment of the cantilever from the fimbriae (Fig. 1D). For several fimbriae at several forces, the extension was measured from this curve and normalized to maximum extension (squares, Fig. 1E). This data was used to fit the parameters for the Worm-Like Chain (WLC) extension of the uncoiled state B and the stretched state C as well as the stretch transition between these states (*k_eq_, x_eq_, l_pB_, l_pC_, x^0^_B_, x°_C_*).

Together this fitting resulted in the parameters shown in the table of Text S1 and dynamic elastic behavior as shown in Fig. 1E. Previous models that did not address the cooperative nature of the uncoiling transition were unable to reproduce the flatness of the main plateau [24]. Previous models that did not include the stretch transition [24] or did not allow for different persistence lengths before and after the stretch transition [25] could not fit the shape of the S-shaped curve (not shown). Thus, our new model was necessary to accurately reproduce the entire range of dynamic stretching data for type 1 fimbriae.

### Yielding elasticity allows bacteria to resist high flow

**Figure 2:**
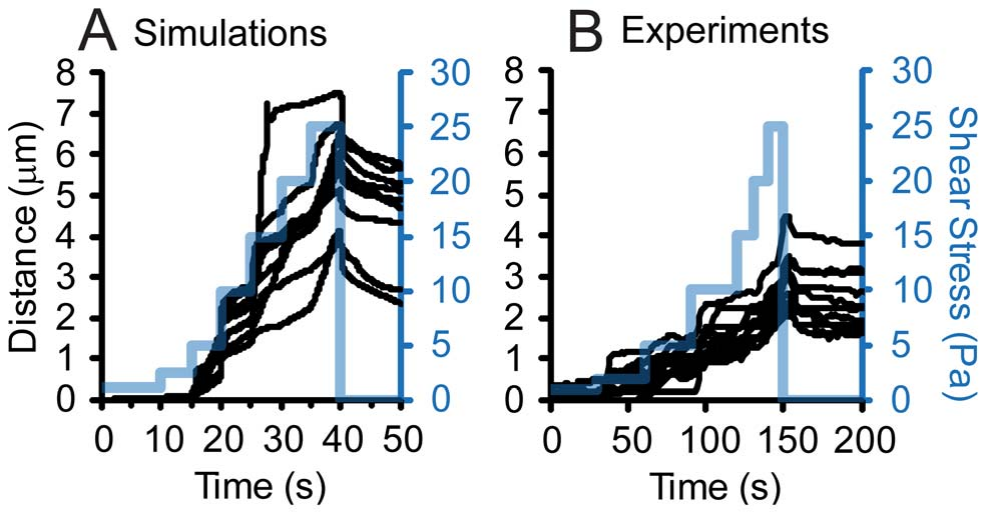
Validation of adhesive dynamics simulations. In simulations (A) and experiments (B), the shear stress was increased stepwise from 1 to 25 Pa, and then dropped back to 0.01 Pa as indicated by the blue lines. The x-positions of several randomly chosen bacteria are shown in black lines.

We incorporated this fimbrial elasticity model into previously developed adhesive dynamics simulations of *E. coli*, and validated the complete simulations by comparison to experimental data. Specifically, the shear stress was stepped up from 1 to 25 Pa in both simulations and experiments, and then dropped to 0.01 Pa. In both cases, the bacteria crept forward as shear increased, and then relaxed backwards when shear decreased, but not back to the original position (Fig. 2). There were small quantitative differences between the bacteria in simulations and experiments; in the simulations, the bacteria moved twice as far, and required slightly higher shear stress to begin moving. However, the relatively close fit is remarkable since there were no free fit parameters for this validation; all 33 simulation parameters were determined independently (Fig. 1 and reference [28]).

**Figure 3:**
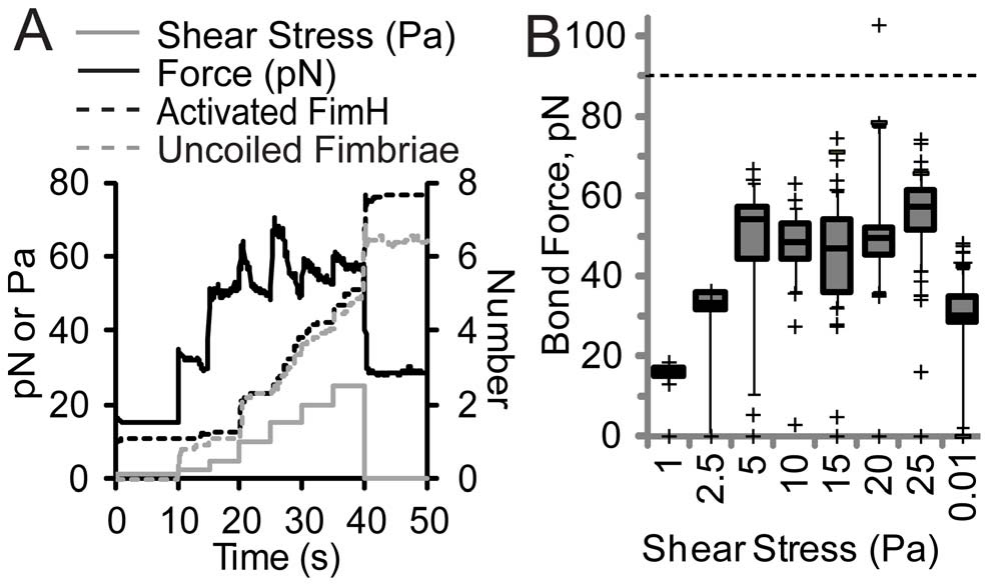
Mechanism of shear-resistance in simulations from Fig. 2. A) The average force per FimH and fimbriae, number of uncoiled fimbriae, and number of activated FimH at each time step (N = 15 simulations). B) The distribution of force on activated FimH bonds is shown 5 seconds after switch to each indicated shear stress. The boxes show the middle two quartiles, the whiskers the 9 to 91% range, and the plus signs the outliers (N = 14 to 98 fimbriae). Activated FimH that were under compression are indicated as zero force since we only consider tensile force here.

The most important observation, observed in both experiments and simulations, is that bacteria never detached, even at 25 Pa, which is higher than most physiological niches. Visual inspection of a typical simulation (e.g. Video S1) revealed the bacterium is anchored in place via one activated FimH bond at low shear, but creeps forward at increased shear, as the anchoring fimbria uncoils, until a second FimH bond is activated, and so on. We analyzed the simulations to quantify these observations. Each time the flow rate was stepped up, the mean force per bond increased suddenly (Fig. 3A), but relaxed back to about 50 pN per bond within seconds, if it had increased above this range (Fig. 3A). This drop in force corresponded to an increase in the number of uncoiled fimbriae and activated FimH bonds (Fig. 3A). Not only did the average force remain at 50 pN as shear increased further, but the distribution of bond forces was narrow (Fig. 3B). It was shown previously that FimH bonds are long-lived between 30 and 70 pN, but break within seconds above 90 pN [41] because of the exponential effects of force, so we consider bonds exposed to over 90 pN force as vulnerable to dissociation. There were almost no vulnerable bonds in these simulations, as indicated by the presence of only one symbol above the dotted line at 90 pN in Fig. 3A. Thus, bacteria in the simulations withstand high shear stress by recruiting more activated bonds and by distributing the force evenly across these bonds.

**Figure 4:**
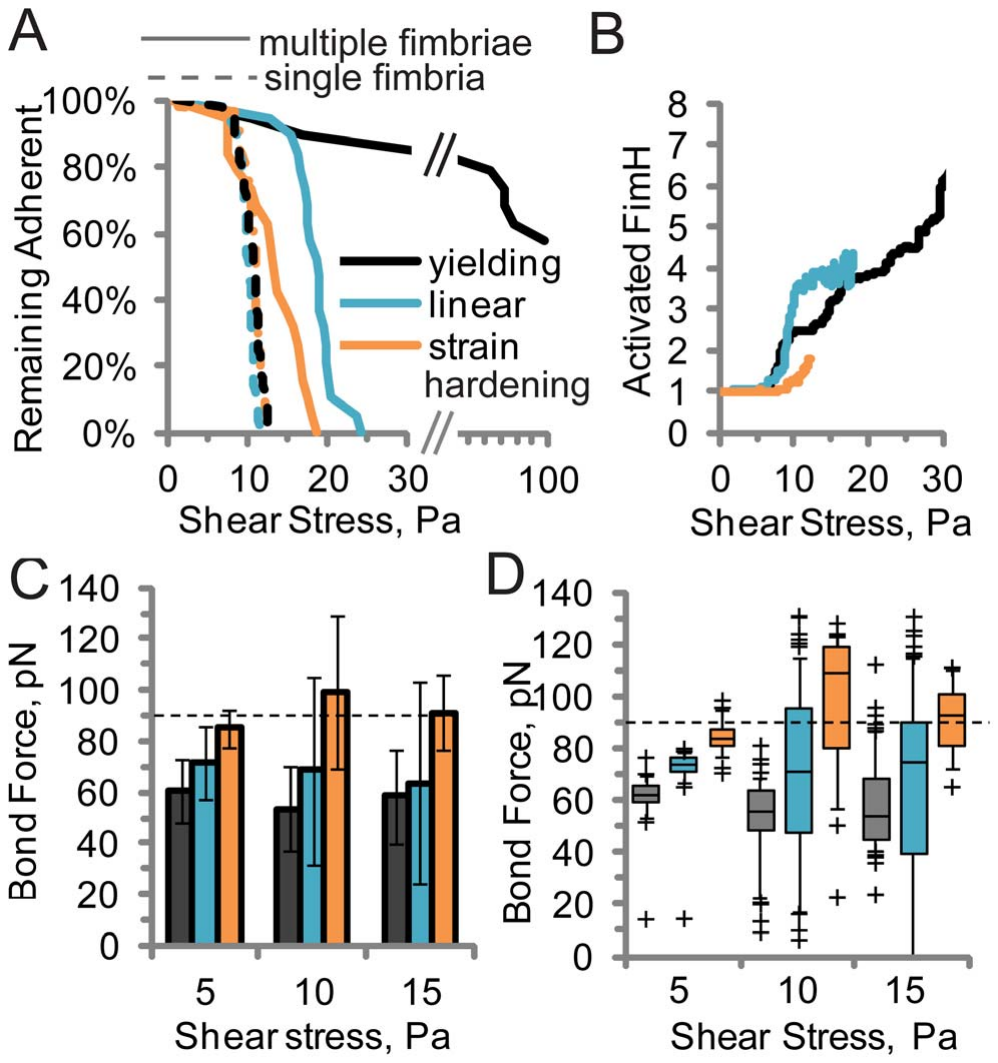
Effect of different fimbrial elastic properties in simulations in which shear stress is increased linearly. A) Percent of simulated bacteria that remain bound (N = 19). B) Average number of activated FimH bonds on remaining bacteria. C) Force per activated FimH. (Error bars  =  SD, N = 12 to 61 fimbriae). D) Distribution of force on activated FimH bonds for same fimbriae as panel C. Box and whisker representation is the same as in Fig. 3. In all panels, black  =  yielding, cyan  =  linear, and orange  =  strain hardening elasticity.

We next asked whether the nonlinear elasticity of the fimbriae was necessary for bacteria to resist high shear stresses. In the simulations, we changed the elastic properties of the fimbriae to model native yielding elasticity, strain-hardening elasticity, or linear elasticity. Shear stress was increased at 1 Pa/s until 100 Pa, or until the bacteria detached. If only one fimbria was allowed to attach (by setting the bond association rate to zero for the unbound fimbriae), then bacteria detached between 10 and 12 Pa for all regardless of the type of tether (Fig. 4A, dashed lines). Allowing multiple fimbriae to bind provided a small improvement for bacteria with strain-hardening tethers, which all detached between 10 and 18 Pa (Video S2), and slightly more improvement to bacteria with linear elastic tethers (Video S3), which detached between 15 and 25 Pa (Fig. 4A). In contrast, bacteria with multiple native yielding tethers withstood much higher shear stress, with very few detaching by 30 Pa, and over 50% remaining bound through 100 Pa (Video S4). Thus, multiple tethers with yielding elasticity were necessary in the simulations to reproduce the ability of bacteria to withstand over 25 Pa, which was observed experimentally.

To understand why the linear and strain hardening elastic tethers were unable to maintain adhesion at high shear stress, we calculated the number of activated bonds per bacterium (Fig. 4B), the mean force per bond (Fig. 4C), and the distribution of bond forces (Fig. 4D). The strain-hardening tethers failed to mediate adhesion at high shear stress because the number of activated bonds remained under two per bacterium, and the average force per bond increased to above 90 pN at and above 10 Pa. In contrast, the linear elastic tethers recruited even more bonds and maintained a similar average force per bond relative to yielding tethers in the same conditions (Fig. 4B). However, the distribution of bond forces for linear elastic tethers was broader, with over one quarter of activated bonds exposed to over 90 pN and thus vulnerable to detachment at and above 10 Pa (Fig. 4D). Since each bacterium had only 3 to 4 activated bonds in these conditions with the linear fimbriae, this means that on average one bond per bacterium breaks rapidly, transferring its load to the remaining bonds, which overloads one of them, and so on. Therefore, linear tethers recruit enough bonds, but fail to protect bacteria from detaching because they do not distribute force evenly between bonds. This demonstrates that the ability to recruit more bonds and distribute force evenly between them, which stabilizes adhesion at high shear stress, requires yielding elastic tethers.

### Elastic yielding allows bacteria to resist variable flow

**Figure 5:**
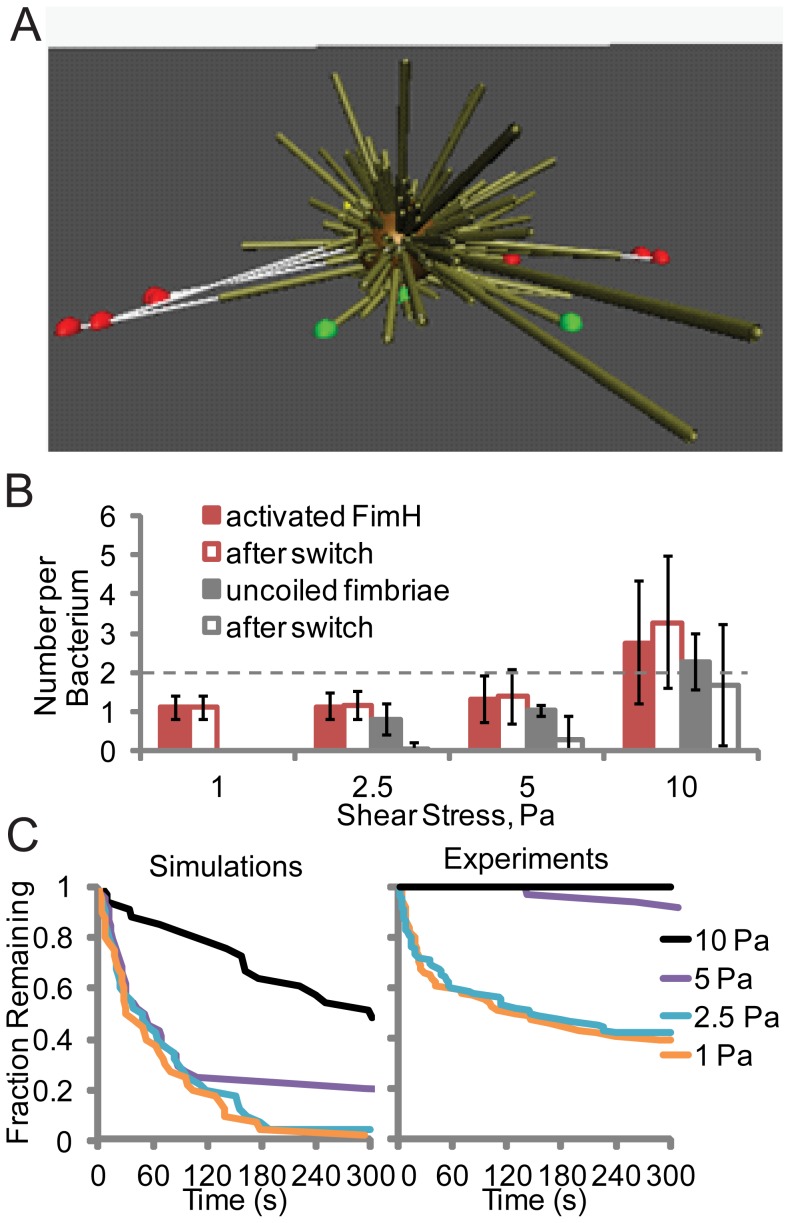
Ability of bacteria to withstand variable flow conditions. A) Cartoon of simulated bacteria suspended between multiple fimbriae after shear stress is decreased from 25 to 0.01 Pa (from left to right). Bonds that are activated and not activated are shown as red and green balls, respectively, and uncoiled sections of fimbriae as thin white lines. B) Number of uncoiled fimbriae and activated bonds at the indicated shear stress before and after a decrease to 0.01 Pa in simulations. (Error bars  =  SD, N = 33 to 44 bacteria). C) Fraction of bacteria remaining bound over time after a decrease from the indicated shear stress to 0.01 Pa in simulations (N = 33 to 44) and experiments (N = 36 to 90).

Bacteria *in vivo* are often exposed to variable shear stress due to intestinal peristalsis or salivary motion. Bacteria binding via FimH catch bonds were shown previously to detach when the flow is turned down from 2 to 0.01 Pa [42], presumably because catch bonds detach at low force. However, in our current study, bacteria relaxed backwards but did not detach when shear stress was dropped from 25 to 0.01 Pa, in both experiments or in simulations (Fig. 2). Surprisingly, the number of activated bonds increased when shear stress dropped to 0.01 Pa (Fig. 3A). Moreover, while the drag force on a bacterium at 0.01 Pa is only 0.2 pN, the force per bond did not drop to near zero, but rather remained tightly distributed around 30 pN (Fig. 3B). Simulations show that the uncoiled fimbriae shorten when shear is decreased, pulling the bacterium backwards, and activating new bonds as the bacterium becomes suspended between partially uncoiled fimbriae pulling in opposite directions (Fig. 5A and Video S1). Since the bacterium is now stationary, the anchoring bond is subjected to the equilibrium uncoiling force (32.2 pN) for all partially uncoiled fimbriae.

We thus predicted that the bacteria would only stay attached at 0.01 Pa in simulations if they were first subjected to enough shear stress to uncoil fimbriae. To test this prediction, bacteria in both simulations and experiments were subjected to 1, 2.5, 5, or 10 Pa, and then dropped to 0.01 Pa. Below 5 Pa, most bacteria had one or fewer uncoiled fimbriae and activated FimH bond (Fig. 5B), and almost none maintained activated bonds after shear was decreased to 0.01 Pa. In contrast, at 10 Pa, most bacteria had 2 or more uncoiled fimbriae and activated bonds (Fig. 5B), and consistently retained activated bonds after shear was decreased. This corresponded to the ability of bacteria to remain attached after shear stress was decreased from 10 Pa but not from 5 Pa or less (Fig. 5C). This supports the idea that uncoiling and recoiling are needed to withstand variable shear stress. Finally, we validated this prediction by performing the same test in experiments (Fig. 5D). Slight quantitative differences were observed, with bacteria in experiments requiring slightly less shear stress for the same behavior, and with a higher fraction failing to detach at low flow. Thus, the experiments validated the prediction that bacteria can withstand a prolonged period at low flow better after being subjected to enough shear stress to uncoil fimbriae.

## Discussion

In this work, we draw our important conclusions from the simulations themselves, so it is essential that they be reliable. We ensure this by using a previously validated model in which almost all parameters were identified independently in cell-free assays, with only two parameters determined by fitting the simulations to cell adhesion data [28]. To add fimbrial uncoiling to this model, we determined all parameters independently by characterizing the elastic properties of individual type 1 fimbriae with atomic force microscopy, and fitting the data with a biophysical model (Fig. 1). Finally, we validated the accuracy of the combined model for cell adhesion by testing predictions of the model (Fig. 2, 5). Since none of the 33 parameters were adjusted to fit the cell adhesion data, minor quantitative discrepancies are expected, such as the higher shear stress required for the same behavior (Fig. 2, 5), and larger distance moved (Fig. 2) in simulations relative to experiments. The first discrepancy suggests that we underestimated the drag coefficient [28]. The second discrepancy suggests that we underestimated the number of fimbriae from the 2D projection in the electron micrographs. These small quantitative differences likely vary from cell to cell and do not affect the conclusions of this paper. The creeping we observe in simulations also resembles the behavior of *E coli* binding through type 1 fimbriae as flow increased in a recent publication [43]. The ability to reproduce a variety of adhesive behaviors with no adjustable parameters provides a high level of certainty to the conclusions drawn from the simulations.

Our major conclusion is that *E. coli* can withstand high shear stress because yielding elastic tethers called fimbriae distribute the drag force equally between multiple bonds (Fig. 3B). To understand why this is important, consider that simple mechanics theory dictates that cell adhesion in flow, like many other conditions, occurs in a peeling manner in which tethers at one edge of the adhesive contact zone are stretched the farthest [11]. While many studies have shown that tethers exhibit some sort of strain-softening, or yielding, viscoelastic behavior [13], [14], [15], [16], the theories developed to address the strength of clusters of bonds during rolling or peeling have assumed that the tethers are linearly elastic, so longer tethers apply proportionally higher force [11], [12]. Since bond lifetimes decrease exponentially with force above a critical value even for catch bonds, the bonds under most force break first, transferring force to the remaining bonds, and causing the cluster of bonds to unzip [7]. Indeed, we observe this behavior in our simulations with linear tethers, which apply a wide range of forces on bonds (Fig. 4D) and peel from the rear until they detach as the drag force is increased (Video S3). Most biological polymers and materials demonstrate nonlinear elastic properties. We show here that strain-hardening materials, which concentrate force even more on the most stretched tethers (Fig. 1, 4D), mediate even weaker adhesion in flow (Fig. 4A). However, many cells have evolved yielding elastic tethers, which provide a constant or nearly constant force independent of extension length (Fig. 1). It is well understood that bond clusters are mechanically stronger when oriented relative to force such that all bonds are stretched equally, rather than oriented so that force can unzip the cluster by stretching them one at a time [7], because the former distributes force better. However, we demonstrate here that yielding tethers can ensure equal force distribution even when bonds are stretched unequally, preventing peeling or unzipping in situations such as cell adhesion in flow. In our study, we assume that each tether has only one FimH, because this is dictated by the structure of type 1 fimbriae [26], [27]. Tethers such as microvilli can have multiple receptors per tether. In these cases, the force per tether may be distributed between multiple receptors, but our conclusions about load sharing between tethers should still apply. Thus, adhesion with yielding tethers is much more robust.

Our results demonstrate that robust adhesion requires the perfect load distribution that is unique to yielding elasticity (Fig 4). However, other previously demonstrated properties of yielding elasticity also benefit cell adhesion. Yielding provides a mechanism for creating long tethers, which reduce the force on a bond when a cell is anchored to a surface via a single tether in flowing fluid [44], although this effect is similar for long tethers with any elastic property, as shown in Fig. 4A (dashed lines). Yielding elastic tethers of all sorts are also usually viscoelastic [16], [45], [46], [47], so that they buffer force on a single bond in variable flow conditions [39], [40], or during dynamic single molecule force spectroscopy [14], [15]. Previous studies have shown that yielding forces are optimized for the catch bonds at their tips [19], [40], [48], which suggests that robust cell adhesion requires not just yielding, but yielding at a force that is appropriate for the mechanical properties of the bond supported by the yielding tether. Our conclusion can also explain previous observations about yielding tethers. Theory and simulations showed that elastic yielding tethers allowed clusters of bonds loaded in parallel to survive much longer than single bonds [37] and experiments showed that long yielding tethers greatly increased the adhesive strength of clusters of bonds between two surfaces [49]. In summary, while yielding elasticity provides many advantages to buffering force on single bonds, we demonstrate here that elastic yielding is most critical to robust adhesion of cells or large bond clusters because it uniquely distributes load equally in a complex environment.

This conclusion may apply to many cell types, because many cells have elastic yielding tethers comprised of alpha helical proteins [50], unfolding domains [51], quaternary helices [43], or membrane tethers [52], all of which yield under force. For example, leukocytes and platelets extend membrane microvilli with selectin or GPIb [13], [53], fibroblasts bind to extensible fibronectin [23] through integrin 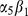, platelets to extensible fibrin [54], bacteria bind through many types of quaternary helical fimbriae that yield [20], [21], [22], [55], and many adhesion proteins like integrins and cadherins are anchored to the cytoskeleton via alpha-helical adaptor proteins that also unfold [50]. Our simulations and experiments used catch bonds, and many of the proteins anchored to yielding tethers also form catch bonds, including P-selectin [1], L-selectin [56], GPIb [3], integrin 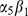 [4], and fibrin knob-hole interactions [57]. Catch bonds and yielding tethers appear to co-evolve to provide the ideal force to optimize catch bond lifetime in order to enable robust binding in high force environments [19]. Catch bonds with yielding tethers (Fig. 3A and 4B), but not catch bonds with other elastic anchors (Fig. 4B) allow the number of activated bonds to increase proportionally to the flow rate, finally providing a mechanism for the ‘automated braking system’ observed previously for leukocytes binding via selectins [58]. Nevertheless, it is unlikely that the importance of yielding tethers is unique to catch bonds, since all catch bonds transition to slip bonds above a critical force, and in our simulations, the ability to distribute force evenly was critical to preventing the failure of FimH bonds in the high-force slip regime. This analysis suggests that yielding elastic tethers may be critical for robust adhesion of a wide range of cells binding through a wide range of receptors.

While the importance of yielding tethers to bond force distribution has not been shown previously for cell adhesion, yielding elasticity has been shown to be important in related fields. Adhesives are weak in a peeling mode, where load is applied so that stress concentrates at one edge, which propagates in a crack as the adhesive fails. This is minimized by soft thin film adhesives that can undergo a plastic deformation to form long yielding fibers that distribute stress equally along multiple fibers in spite of their difference in length. Because this deformation is irreversible, the thin film adhesives are weakened by this process, and could be improved by the development of a bio-inspired adhesive material that exhibits fully reversible viscoelastic yielding, like the yielding biological tethers described above. Yielding elasticity has also been demonstrated in fibers that make up certain biological materials, such as fibrin clots [54] and the spectrin network in red blood cell membranes [59]. These materials are resistant to tearing because yielding fibers prevent stress concentration. Thus, thin film adhesives, biological materials and cell adhesion are all strengthened by yielding fibers that prevent stress concentration and crack propagation. However, cell adhesion provides a new level of elegance, as the yielding force of the fibers must be optimized for the lifetime of the adhesive bond.

## Materials and Methods

### AFM experiments

AFM experiments were conducted with an Asylum MFP3D AFM to determine the dynamic behavior of fimbriae in response to force. Olympus Biolever cantilevers were incubated with RNaseB (a naturally mannosylated protein) and surfaces with type 1 fimbriae using direct nonspecific adsorption, essentially as previously described [49]. Force pulls were controlled with a custom written script that allows back-and-forth movements at speeds from 0.1–10 µm/s. Plateau forces were determined by averaging at least 23 separate pulls from 2–4 experiments performed on different days with different cantilevers, except for the condition of recoiling at 10 µm/s, for which only 8 pulls were performed. All experiments were conducted in Phosphate Buffered Saline with 0.2% Bovine Serum Albumin (PBS/BSA) to prevent nonspecific adhesion. We have shown previously that proteins incubated in this manner remain adherent under much higher forces than 150 pN [2], and that adhesive strength is maintained even after hundreds of pulls on the same surface-immobilized fimbriae [49], so it is safe to assume that the proteins remain attached to the surface and cantilever during our experiments. Thus, the observed yielding behavior is not due to an experimental artifact.

### Flow chamber experiments

Flow chamber experiments were performed as previously described [42]. Briefly, a bolus of *E. coli* expressing KB-91 FimH and K12 fimbrial shafts was introduced at a moderate shear stress (0.1–0.3 Pa) to allow bacteria to accumulate and then the shear was increased to 1 Pa to induce predominately stationary adhesion and to wash out unbound bacteria. The shear was then increased or decreased as indicated in each figure. Time-lapse videos were taken at 1 frame per second and analyzed to quantify cell position and detachment. In some experiments, a second syringe pump was used in parallel with the first to deliver a soluble inhibitor with minimal disruption to the system.

### Simulations

Simulations were performed as previously described [28] except that the fimbrial uncoiling model was added. Briefly, the 3D simulations model the interaction of a fimbrial-coated spherical cell with a mannose-coated planar surface in a laminar fluid flow. The tip of each fimbria represents a single FimH adhesin which can stochastically form and break bonds with the surface according to the two-state allosteric catch bond model [60]. Fimbriae can stretch, bend and buckle due to linear elastic properties [28], or uncoil and recoil with higher tension using the model described below. Simulations start with a single fimbria bound to the surface in the high-affinity state representing a bacterium that has just transitioned to stationary adhesion as in the experiments. In simulations with only a single fimbriae, the fimbriae was always set to 1 µm in length to remove differences that result from varied fimbrial lengths. In simulations with multiple fimbriae, bacteria were surrounded by 186 randomly distributed fimbriae with an average length of 0.572 µm around an exponential distribution [61].

All fimbrial models had the same length distribution as the native fimbriae. The native yielding fimbriae were modeled with a three state model in which each subunit can be in state A (fully coiled), state B (uncoiled), or state C (uncoiled and stretched), with transitions allowed between A and B and between B and C, as illustrated in Fig. 1B. The subunits in state A form a contiguous segment, since uncoiling is a cooperative phase transition that only occurs at the edge of the helical coil, as indicated by the flat uncoiling transition in Fig. 1D and 1E. However, the subunits in states B and C form noncontiguous segments because the stretch transition occurs independently for any uncoiled subunit, as indicated by the sloped stretch transition in Fig. 1D and 1E. The number of subunits in each segment is determined by force-dependent transitions between the states, according to the Bell model. The A segment was modeled as a spring, and the B and C segments were modeled as worm-like chains with different persistence lengths. The total length of the fimbriae is the sum of the lengths of the three segments. All parameters were fit to AFM data. The linear elastic tethers were modeled by disallowing all uncoiling, so that the native linear elasticity is always in effect. The strain-hardening tethers were modeled as if the uncoiling occurs at negligible force, so that they elongated beyond their native rod length under force with the WLC model, using the parameters for segment B. A complete description of the uncoiling model is provided in Text S1.

## Supporting Information

Txt S1 **Method for simulating fimbrial yielding.**
(DOCX)

Video S1 **Bacteria resist detachment as shear stress is increased stepwise.** A single bacterium was simulated as in Figure 2A, with fluid moving from left to right as shear stress changes as indicated. Bonds that are activated and not activated are shown as red and green balls, respectively. Native and uncoiled sections of fimbriae are shown as thick gold and thin black lines, respectively.
(AVI)

Video S2 **Strain-hardening elasticity.** A bacterium with strain-hardening tethers is simulated as in Figure 4, with fluid moving from left to right as shear stress increases as indicated. Note that the bacterium move forward long distances each time a bond breaks, because the soft tethers elongate long distances. However, by 17 Pa, new bonds do not form in time after bonds break, and the bacterium detaches. Bonds that are activated and not activated are shown as red and green balls, respectively. Native and uncoiled sections of fimbriae are shown as thick gold and thin black lines, respectively.
(AVI)

Video S3 **Linear elasticity.** A bacterium with linear elastic tethers is simulated as in Figure 4, with fluid moving from left to right as shear stress increases as indicated. Note that the bacterium moves forward short distances each time a bond breaks, because the stiff tethers elongate very little. However, by 20 Pa, new bonds do not form in time after bonds break, and the bacterium detaches. Bonds that are activated and not activated are shown as red and green balls, respectively. Native and uncoiled sections of fimbriae are shown as thick gold and thin black lines, respectively.
(AVI)

Video S4 **Yielding elasticity.** A bacterium with native yielding tethers is simulated as in Figure 4, with fluid moving from left to right as shear stress increases as indicated. Note that the number of activated bonds increases with shear stress, so that the bacterium does not detach. Bonds that are activated and not activated are shown as red and green balls, respectively. Native and uncoiled sections of fimbriae are shown as thick gold and thin black lines, respectively.
(AVI)
