# Supplementary material for: Yielding Elastic Tethers Stabilize Robust Cell Adhesion
Source: PLoS Comput Biol. 2014 Dec 4;10(12):e1003971. doi: 10.1371/journal.pcbi.1003971 (PMC4256016; doi:10.1371/journal.pcbi.1003971)
Supplement: Txt S1 — Method for simulating fimbrial yielding. (DOCX) [file pcbi.1003971.s001.docx]

# Text S1. Method for Simulating Fimbrial Yielding

*Overview*

Within each time step in our simulations, the positions of the fimbrial tips and attachment points to the bacterium are known for each bound fimbria. This defines the current length of a fimbria leading to either a tensile or compression force being applied to the adhesive bond and the bacterium. What this force is for a fimbria at a given percentage of its unstressed length must then be calculated. Unlike our previous paper, the individual FimA subunits comprising the fimbrial shaft are incorporated into the model. Subunits can be in one of three possible states: fully coiled (A), uncoiled but still coupled to an adjacent FimA (B), or uncoiled and separated from adjacent subunits (C) (schematically illustrated in Figure S1). Subunits can transition between the three states with rates dependent on the applied force. The total length of the fimbriae *L_T_* is distributed between the overall lengths of the subunits in each state such that *L_T_* = *L_A_* + *L_B_* + *L_C_*. The length of each of these segments in turn depends on the number of subunits in that segment as well as the overall force on the fimbriae.

Segment A is modeled as a Hookean spring while segments B and C are modeled as worm-like chains (WLC). Within a time step, for a given overall fimbrial length and number of subunits in each state, the force on a fimbria is calculated by setting the force within the coiled segment A equal to the force within the combined uncoiled segments B and C (as described in detail in the force modeling section below). This resultant force is used in turn to calculate the transitions between the coiled and uncoiled subunits and within the uncoiled subunits (as described in detail in the dynamics of fimbrial uncoiling section below). While this is an iterative process, the time step in the simulations (0.5 µs) is sufficiently short to allow a single calculation of the force and subunit transitions at each time step.

|   $L_{C}^{o}$  $L_{B}^{o}$  $L_{A}^{o}$ |
| --- |
| **Figure S1.** Schematic of a partially uncoiled fimbria. The subunits of a fimbria are considered to be in 1 of 3 states: A, B, or C. Subunits in A whose force response is modeled as a Hookean spring have an equilibrium length $L_{A}^{o}$. Subunits in B and C which are modeled as WLCs have contour lengths of $L_{B}^{o}$ and $L_{C}^{o}$ respectively. |

*Force modeling*

The coiled segment A of a fimbria is modeled as a Hookean spring supporting a force *F* of *k*(L_A_-*$L_{A}^{o}N_{A}$*)* where *k* is the effective spring constant of a coiled fimbria, $L_{A}^{o}$ is the equilibrium length of a single subunit, and *N_A_* is the number of subunits in the coiled state. *k* was measured in AFM experiments and found to be invariant of fimbrial length implying that the linkage to the cell and/or the fimbrial tip were likely dominant (6). This is not surprising given that a coiled segment (1000s of subunits in series) is expected to be an order of magnitude stiffer than the measured *k* based on the general mechanics of proteins (30).

The uncoiled segments of the fimbriae (states B and C) are modeled as WLCs supporting a force *F* of $\frac{k_{B}T}{l_{pB,C}}\left[ \frac{1}{4}\left( 1-\frac{L_{B,C}}{L_{clB,C}} \right)^{-2}-\frac{1}{4}+\frac{L_{B,C}}{L_{clB,C}} \right]$ where *l_pB,C_* is the persistence length of the uncoiled fimbriae and *L_clB,C_*  is the contour length (maximum extension). The contour length is calculated as the number of subunits (*N_B_* or *N_C_*) multiplied by the maximum extension of a single subunit ($L_{B}^{o}$ or $L_{C}^{o}$). Because the segments are in series, the force in each segment is equal:

$k*\left( L_{A}-L_{A}^{o}N_{A} \right) =\frac{k_{B}T}{l_{pB}}\left[ \frac{1}{4}\left( 1-\frac{L_{B}}{L_{clB}} \right)^{-2}-\frac{1}{4}+\frac{L_{B}}{L_{clB}} \right]=\frac{k_{B}T}{l_{pC}}\left[ \frac{1}{4}\left( 1-\frac{L_{C}}{L_{clC}} \right)^{-2}-\frac{1}{4}+\frac{L_{C}}{L_{clC}} \right]$.

However in order to be more efficient in simulations, we lumped states B and C together into a single WLC with an effective persistence length *L_peff_* that depends on the number of subunits in each of the two states. An analytical solution for the effective persistence length of two WLCs in series could not be found. Therefore, a numerical solution was found by determining the ratio of *N_B_* and *N_C_* and the effective persistence length of the combined WLCs at given forces. Then, a polynomial equation was fit to the relationship between the fraction of subunits in B vs C and L_peff_ and used in simulations to determine L_peff_ at each time step given the current *N_B_*/*N_C_* ratio. The simplified equation, taking into account that *L_B_* + *L_C_* = *L_T_* - *L_A_*, then becomes

$$k*\left( L_{A}-L_{A}^{o}N_{A} \right) =\frac{k_{B}T}{l_{peff}}\left[ \frac{1}{4}\left( 1-\frac{L_{T}-L_{A}}{L_{clB}+L_{clC}} \right)^{-2}-\frac{1}{4}+\frac{L_{T}-L_{A}}{L_{clB}+L_{clC}} \right]$$

which can be solved for *L_A_* and thus the force on the fimbriae.

*Dynamics of fimbrial uncoiling*

The A↔B and B↔C transitions of the FimA subunits are modeled with two distinctly different force dependent processes as described in the following sections.

State A to B transition

The transition rates between states A and B are given by

$$k_{AB}=k_{AB}^{o}\exp\left( \frac{x_{AB}F}{k_{b}T} \right)$$

$$k_{BA}=k_{BA}^{o}\exp\left( \frac{x_{BA}F}{k_{b}T} \right)$$

where $k_{AB}^{o}$ and $k_{BA}^{o}$ are the unstressed transition rates, $x_{AB}$ and $x_{BA}$ are the transition distances, *F* is the force on the bond, *k_b_* is the Boltzmann constant, and *T* is the temperature. Due to the larger number of interactions between subunits within the fimbrial coil compared to the interactions of the last subunit of the coil, transitions are only allowed sequentially from one exposed end making the transition rates independent of subunit number. A deterministic model is used for the transitions instead of a stochastic one as done in Bjornham et al (22). This allows the simulations to be more efficient while still reproducing the force extension behavior of fimbriae. The number of subunits in a coiled or uncoiled state (grouping B and C together in the uncoiled state) is determined using the following equations:

$$N_{A}=k_{BA}t-k_{AB}t+N_{A}^{o}$$

$$N_{BC}={-k}_{BA}t+k_{AB}t+N_{BC}^{o}.$$

These equations are the solution to the coupled differential equations:

$$\frac{dN_{A}}{dt}=k_{BA}-k_{AB}$$

$$\frac{dN_{BC}}{dt}={-k}_{BA}+k_{AB}$$

with initial conditions $N_{A}^{o}$ and $N_{BC}^{o}$.

The equilibrium uncoiling force refers to the force at which the fimbriae neither lengthen nor shorten, so the uncoiling and recoiling rate are the same: $k_{AB}=k_{BA}$, or

$$k_{AB}^{o}\exp\left( \frac{x_{AB}F}{k_{b}T} \right)=k_{BA}^{o}\exp\left( \frac{x_{BA}F}{k_{b}T} \right)$$

This occurs when $F=\frac{k_{b}T}{x_{AB}-x_{BA}}\ln\left( \frac{k_{BA}}{k_{AB}} \right)$, or F = 31.2 pN.

State B to C transition

As noted before, the response during region III of fimbrial uncoiling exhibits no hysteresis for velocities at least from 0.1-10 µm/s indicating that the process is in equilibrium. The B↔C transition responsible for this behavior can therefore by modeled with a single rate constant *k_eq_* given by

$$k_{eq}=k_{eq}^{o}exp\left( \frac{{-x}_{eq}F}{k_{b}T} \right)$$

where $k_{eq}^{o}$ is the unstressed rate and $x_{eq}$ is the transition distance. The number of subunits in each of the two states (*N_B_* and *N_C_*) is determined by this rate and the total number of uncoiled subunits *N_BC_*:

$$N_{B}=\frac{{k_{eq}N}_{BC}}{1+k_{eq}}$$

$$N_{C}=\frac{N_{BC}}{1+k_{eq}}.$$

Implementation of the state B to C transition

The instantaneous changes allowed by the equilibrium equations described above can cause instabilities in simulations. The instabilities could potentially be alleviated by decreasing the time step which would make the simulations more expensive. Alternatively the transitions could be damped, but the damping factor would be rather arbitrary and seemed to require different damping factors at different stages of uncoiling in initial efforts.

Our solution, even though the B↔C transition appears to be at equilibrium, is to explicitly model the transitions between states. Unlike the A↔B transition, the B↔C transition is dependent on the number of subunits because transitions can occur anywhere within the uncoiled segment. Transitions rates are given by:

$$k_{BC}=k_{BC}^{o}\exp\left( \frac{x_{BC}f}{k_{b}T} \right)$$

$$k_{CB}=k_{CB}^{o}\exp\left( \frac{x_{CB}f}{k_{b}T} \right).$$

The number of subunits in each state is determined by:

$$N_{B}=\frac{\left\{ {k_{CB}N}_{U}-\left( {k_{CB}N}_{C}^{o}-k_{BC}N_{B}^{o} \right)exp\left[ -\left( k_{CB}+k_{BC} \right)t \right] \right\}}{k_{CB}+k_{BC}}$$

$$N_{C}=\frac{\left\{ {k_{BC}N}_{U}-\left( {k_{CB}N}_{C}^{o}-k_{BC}N_{B}^{o} \right)exp\left[ -\left( k_{CB}+k_{BC} \right)t \right] \right\}}{k_{CB}+k_{BC}}.$$

These equations are the solution to the coupled differential equations:

$$\frac{dN_{B}}{dt}=k_{CB}N_{C}-k_{AB}N_{B}$$

$$\frac{dN_{C}}{dt}={-k}_{CB}N_{C}+k_{AB}N_{B}$$

with initial conditions $N_{B}^{o}$ and $N_{C}^{o}$.

### Parameter fitting

The 10 unknown parameters required for the equations above were determined by fitting simulations to experimental AFM data in Matlab.

The four parameters required to describe the uncoil/recoil transition (k_AB_ k_BA_, x_AB_, x_BA_) were determined by conducting AFM force spectroscopy experiments at velocities from 0.1-10 µm/s. Because the transition is not at equilibrium at these speeds, different plateau forces were observed during approach and retraction. Plateau forces were determined by averaging at least 20 separate pulls from 2-4 experiments performed on different days with different cantilevers. As described in Forero et al (8), the expected velocity of uncoiling or recoiling at a given force can be calculated using

$$v=\Delta L_{AB}\left( k_{AB}-k_{BA} \right)$$

where $\Delta L_{AB}$is the length change at uncoiling (5 nm). The set of four parameters that best reproduced the uncoiling and recoiling velocities found in experiments at given forces was found by error minimization.

Following this optimization, the remaining 6 parameters (k_eq_, x_eq_, *l_pB_*, *l_pC_*, $L_{B}^{o}$, and $L_{C}^{o}$) were estimated by fitting the region III behavior to the average behavior obtained in AFM experiments. A series of measurements of the force versus distance were taken during region III at a variety of pulling velocities. All fimbriae were between 2-8 µm in uncoiled length. To account for the differences in length, the x-distance measurements were scaled by dividing the values by the distance when the force reached 150 pN. A set of pulls for each condition was performed without fimbriae present to serve as a negative control and to establish any hydrodynamic effects so that they could be accounted for in the data analysis.

Table S1 **Parameters for Fimbrial Elastic Model**

| **Parameter** | **Value** | **Method** |
| --- | --- | --- |
| k_AB_ | 0.069 s^-1^ | Plateau force |
| k_BA_ | 4.3x10^6^ s^-1^ | Plateau force |
| x_AB_ | 0.461 nm | Plateau force |
| x_BA_ | -1.9 nm | Plateau force |
| k_eqBC_ | 2.63x10^5^ s^-1^ | S-curve fit |
| x_eqBC_ | 0.64 nm | S-curve fit |
| L_pB_ | 3.35 nm | S-curve fit |
| L_pC_ | 0.516 nm | S-curve fit |
| k^*^ | 2.06 pN/nm | ([24](#_ENREF_24)) |
| $x_{A}^{o}$^**^ | 0.7 nm | ([19](#_ENREF_19)) |
| $x_{B}^{o}$ | 6.1 nm | S-curve fit |
| $x_{C}^{o}$ | 8.2 nm | S-curve fit |
